# Genotypic and phenotypic characteristics of *Streptococcus pneumoniae* from community-acquired pneumonia patients and healthy asymptomatic participants in Sichuan province, China

**DOI:** 10.1186/s12879-021-06737-w

**Published:** 2021-10-01

**Authors:** Shihui Peng, Hongyu Ren, Jianping Deng, Na Zhao, Yinan Li, Ming Li, Qiwu Yuan, Zhengdong Zhang, Longze Luo, Linzi Zeng, Bin Wang, Nianli Zou, Changguo Gu, Xin Huang, Zheng Liao, Shenen Chen, Haiying Chen, Qun Li, Tian Qin

**Affiliations:** 1 The Collaboration Unit for Field Epidemiology of State Key Laboratory for Infectious Disease Prevention and Control, Jiangxi Provincial Key Laboratory of Animal-Origin and Vector-Borne Diseases, Nanchang Center for Disease Control and Prevention, Nanchang, People’s Republic of China; 2 State Key Laboratory for Infectious Disease Prevention and Control, National Institute for Communicable Disease Control and Prevention, Chinese Center for Disease Control and Prevention, No. 155, Chang Bai Road, ChangPing District, Beijing, 102206 People’s Republic of China; 3 Zigong Center for Disease Control and Prevention, Zigong, People’s Republic of China; 4 Chengdu Center for Disease Control and Prevention, Chengdu, People’s Republic of China; 5 Sichuan Center for Disease Control and Prevention, Chengdu, People’s Republic of China; 6 The Fifth People’s Hospital of Chengdu, Chengdu, People’s Republic of China

**Keywords:** *Streptococcus pneumoniae*, Serotype, Antimicrobial susceptibility, Virulence gene, Molecular epidemiology

## Abstract

**Background:**

*Streptococcus pneumoniae* (*S. pneumoniae*) is the common cause of community-acquired pneumonia (CAP) and is also found in the upper respiratory tract of healthy people. Hence, the study aimed to compare the serotypes, virulence/pili genes, and antibiotic susceptibility of *S. pneumoniae* from healthy asymptomatic participants and CAP patients.

**Methods:**

*Streptococcus pneumoniae* were retrospectively collected from health asymptomatic participants and CAP patients in Sichuan, China. The serotypes were tested by multiplex polymerase chain reaction (PCR) or Quellung reaction. Antibiotic susceptibility testing was performed using the broth microdilution method. The molecular epidemiology of *S. pneumoniae* was analyzed by multilocus sequence typing (MLST). Additionally, the presence of virulence/pili genes were detected using PCR.

**Results:**

A total of 83 pneumococcal isolates were collected in the current study. Of these, 52 and 31 isolates were from healthy asymptomatic participants and CAP patients, respectively. Most of *S. pneumoniae* were resistant to erythromycin (ERY), clindamycin (CLI), tetracycline (TET) and trimethoprim-sulfamethoxazole (SXT). 90.4% isolates were classified as multidrug resistant (MDR). The predominant serotypes were 3, 19F and 19A in the CAP carriers, whereas 3, 6 and 19F were the main serotypes among the asymptomatic carriers. The overall coverage rates of pneumococcal conjugate vaccine (PCV) 10 and PCV13 serotypes were 34.9% and 66.3%, respectively. The predominant sequence types (STs) were ST271, ST320, and ST3397. There were significant differences in some resistance and virulence characteristics between CAP patients and asymptomatic carriers. Additionally, clonal complex (CC) 271 strains had higher percentage in resistance to cefuroxime (CXM) and cefotaxime (CEF), meropenem (MER) and cefepime (CFP), which mainly carried the *rlrA* and *sipA* genes.

**Conclusions:**

High coverage rate of PCV13 and high prevalence of MDR indicated the necessity to expand immunization with PCV13 and rationally use the antibiotics in Sichuan, China. Importantly, long-term surveillance should be conducted to assess effectiveness brought by vaccines. Our findings may supply new guidance for developing new pneumococcal vaccines.

## Background

Community-acquired pneumonia (CAP) is an infection of lung parenchyma that occurs in persons outside the hospital, which has high rates of morbidity and mortality, especially in children and elderly [1, 2]. Among the pathogens, *Streptococcus pneumoniae* (*S. pneumoniae*) is considered as a major cause of CAP [3]. Since the first detection of antibiotic-resistant *S. pneumoniae* in 1977, multidrug-resistant (MDR) *S. pneumoniae* has appeared in various countries [4–6]. Additionally, previous study has stated that the multidrug resistance of *S. pneumoniae* in China was the highest among the 11 Asian countries [7], indicating the urgent needs for controlling antimicrobial resistance.

It is well known that analysis of the molecular characteristics exerts a key role in clinical treatment. To date, over 90 serotypes of *S. pneumoniae* were identified [8]. The diversity is based on variation in structure of the repeating units of capsular polysaccharide [9]. Additionally, capsular polysaccharides as virulence factors, are the basis of *S. pneumoniae* vaccines, including the 7-valent pneumococcal conjugate vaccine (PCV7), the 10-valent PCV (PCV10), and the 13-valent PCV (PCV13) [10]. Although PCV7 vaccine is introduced in 2008, the immunization rate of PCV7 was less than 10% because it had not been included into National Immunization Program in China. Especially among migrant children, they are not largely vaccinated [11]. In 2017, PCV13 vaccine is available in big cities of China, but this vaccine is given only on own expense. Additionally, a previous study has stated that serotype distribution varies in Asian countries, and non-PCV serotypes have emerged as well [12]. Therefore, monitoring local serotypes can prevent the occurrence of pneumococcal disease and provide the guidance for developing new pneumococcal vaccines.

*Streptococcus pneumoniae* normally colonizes the upper respiratory tract of humans, and previous studies have shown the prevalence of *S. pneumoniae* carriage of 16.6% for healthy children in Shanghai, and nearly 70.0% for healthy children in Xinjiang, China [13, 14]. In the pathogenesis of pneumococcal disease, nasopharyngeal colonization is the necessary first step [15], suggesting that exploration of *S. pneumoniae* characteristics in asymptomatic carriers is warranted. Although epidemiology studies of *S. pneumoniae* from patients with CAP and IPD have been conducted in China [16, 17], limited data are available for the genotypic characteristics of *S. pneumoniae* from asymptomatic participants and CAP patients.

The study aimed to explore the genotypic and phenotypic characteristics of *S. pneumoniae* from asymptomatic participants and CAP patients in Sichuan, China, which may provide new guidance for PCVs coverage and developing new pneumococcal vaccines.

## Methods

### Study population

An observational and retrospective study to collect *S. pneumoniae* between January 2018 and December 2018 was conducted. The study was approved by the ethical committee of Chinese Center for Disease Control and Prevention. 600 patients diagnosed as CAP in the Fifth People’s Hospital of Chengdu and 598 healthy asymptomatic participants attending the routine health examination in Zigong Center for Disease Control and Prevention were included.

The inclusion criteria for CAP patients were patients (1) who were hospitalized due to diagnosis with CAP; (2) aged > 1 year old; (3) with *S. pneumoniae* isolated from blood specimens at hospital admission; (4) who did not vaccinate PCVs.

The inclusion criteria for asymptomatic participants were people (1) who were healthy; (2) aged > 1 year old; (3) who had no respiratory disease in this and previous physical examinations and no respiratory symptoms for nearly one month; (3) who lived in Zigong over 1 year; (4) who did not take any medication in the past one month prior to the study; (5) who did not vaccinate PCVs. The people were excluded if they had wounded nose (nasal trauma and injuries), or received antibiotics within the past one month. Oropharynx samples were collected from healthy asymptomatic participants using nylon-tipped swabs.

### Microbiological studies

All specimens were transported to the department of clinical microbiology within 2 h. Following, specimens were cultured on Columbia blood agar with 5% sheep blood (OXOID, Basingstok, UK) and incubated at 37 °C for 24–48 h. *S. pneumoniae* was identified by colony morphology, hemolytic reaction, Gram staining, optochin susceptibility, and bile solubility testing.

### Determination of capsular types

The capsular types were determined by multiplex polymerase chain reaction (PCR) as previously described [18]. If isolates could not be typed, standard Quellung reaction with pneumococcal typing antisera (Statens Serum Institut, Copenhagen, Denmark) was further applied [19]. Serotypes that could not be identified by multiplex PCR and Quellung reaction were classified as non-typeable (NT).

### Multilocus sequence typing

*Serotypeable S. pneumoniae isolates* were investigated by Multilocus sequence typing (MLST) analysis. In this method, the internal fragments of seven housekeeping genes (*aroE, gdh, gki, recP, spi, xpt,* and *ddl*) were amplified by PCR as previously described [20]. The sequence types (STs) were obtained by sequencing, followed by submitting the sequences to the *S. pneumoniae* MLST database (http://pubmlst.org/spneumoniae/) for identification.

### Antimicrobial susceptibility test

According to Clinical and Laboratory Standards Institute (CLSI) M100-S28 documents [21], the minimum inhibitory concentrations (MICs) of *S. pneumoniae* against 17 antimicrobial agents were determined by automated system (SCENKER, Liaocheng, China). MICs of erythromycin (ERY) and clindamycin (CLI) were determined using MIC strips in all ERY/CLI non-susceptible isolates (OXOID, Basingstok, UK). *S. pneumoniae* ATCC 49619 was used as the control strain. Isolates that were resistant to three or more classes of antimicrobial agents were defined as MDR *S. pneumoniae*.

### Description and detection of virulence genes

Pneumolysin and autolysin are encoded by the *ply* and *lytA* genes, respectively, which can enhance the virulence of *S. pneumoniae*. Additionally, pneumolysin binds membrane cholesterol and forms transmembrane pores that lead to cell lysis [22]. Pneumococcal surface protein A is one of surface-exposed choline binding proteins, which is encoded by *pspA* gene and presents almost in all pneumococcal isolates [23]. The *pavA* gene of *S. pneumoniae* encodes a fibronectin-binding protein, which is crucial for virulence [24]. The *cbpA* and *cbpG* genes encode choline binding proteins that promote the colonization of the nasopharynx [25].

The virulence genes (*ply, lytA, pspA, pavA, cbpA, cbpG*) and pilus genes (*rlrA and sipA*) of *S. pneumoniae* were amplified using PCR. The primers, PCR reactions and conditions were used as previously described [26]. The 100-bp plus DNA ladder marker (Takara, Dalian, China) was used for estimating molecular weight.

### Statistical analysis

Categorical variables were analyzed by the Chi-square or Fisher’s exact tests using the software SPSS22. P-values < 0.05 were considered significant. BioNumerics software (Version 7.1, Applied Maths, Kortrijk, Belgium) was used to create a cluster tree and minimum spanning trees (MST), based on the allelic profiles. In MST, a clonal complex (CC) was formed by STs with six of seven MLST alleles in common and at least three STs; the founder ST was defined as the ST with the highest number of single-locus variants (SLVs); single genotypes that did not correspond to any clone groups were defined as singletons. The size of each circle indicated the number of strains of that particular type.

## Results

### Characteristics of study population

As shown in Table 1, the median ages of asymptomatic participants overall and CAP patients overall were 27.5 and 28.5 years, respectively (P = 0.727). There were no statistical differences in the distributions of age and gender between two groups (P = 0.727, P = 0.088).

**Table 1:** Characteristics of study population

Characteristics	Asymptomatic participants overall (n = 598)	CAP patients overall (n = 600)	P value	Asymptomatic carriers (n = 52)	CAP carriers (n = 31)	P value
Age, median (IQR)	27.5 (10, 58)	28.5 (5, 54)	0.727	5 (4, 8)	67 (45, 73)	0.000
Age group (n, %)			0.727			0.000
≤ 5 years	158 (26.4)	107 (17.8)	–	33 (63.5)	7 (22.6)	–
6–14 years	73 (12.2)	97 (16.2)	–	13 (25.0)	8 (25.8)	–
15–24 years	57 (9.5)	85 (14.2)	–	4 (7.7)	1 (3.2)	–
25–44 years	103 (17.2)	52 (8.7)	–	0 (0.0)	3 (9.7)	–
45–64 years	109 (18.2)	179 (29.8)	–	1 (1.9)	4 (12.9)	–
> 65 years	98 (16.5)	80 (13.3)	–	1 (1.9)	8 (25.8)	–
Gender (n, %)			0.088			0.797
Male	368 (61.5)	387 (64.5)	–	27 (51.9)	17 (54.8)	–
Female	230 (38.5)	213 (35.5)	–	25 (48.1)	14 (45.2)	–

A total of 83 pneumococcal isolates were obtained from 598 healthy asymptomatic participants and 600 CAP patients. Of these, 52 isolates (8.7%) were collected from asymptomatic participants, among which 27 were male (51.9%) and 25 were female (49.1%), respectively. The median age of asymptomatic carriers was 5 years. Among the asymptomatic carriers, *S. pneumoniae* were mostly from children ≤ 5 years of age (63.5%). Additionally, 31 *S. pneumoniae* (5.2%) were obtained from CAP patients. 17 isolates of *S. pneumonia* were from male (54.8%) and 14 from female (45.2%). The median ages of CAP patients were 67 years. There was no statistical difference in gender distribution between CAP carriers and asymptomatic carriers (P = 0.797), but there was significant difference in age distribution (P = 0.000). The positive rate for pneumococcus in CAP patients was significantly different from asymptomatic participants (P = 0.000).

### Distribution of capsular types and PCVs coverage

As shown in Table 2, there were 21 capsular types, as well as 5 NT isolates detected among 83 *S. pneumoniae*. Among them, 16 serotypes and 5 NT isolates were identified in asymptomatic carriers group, and the major serotypes were 3 (23.1%), 6 (17.3%), and 19F (7.7%). For isolates from CAP carriers, there were 9, 7, and 3 cases of serotypes 3, 19F, and 19A; 2 cases each of serotypes 8, 23A, and 23F; and 1 case each of serotypes 4, 6, 7, 14, 20, and 35A/C. The overall coverage rates of PCV10 and PCV13 were 34.9% and 66.3%, respectively. The coverage rate of PCV13 in the CAP carriers group (80.6%) was higher than that of asymptomatic carriers group (57.7%) (P = 0.03). Furthermore, rates of PCV13/non-PCV7 serotypes in the CAP carriers and asymptomatic carriers groups were 41.9% and 26.9%, respectively (P = 0.16). Serotypes 11, 13, 15B/C, 16F, 18, 22, 23B, 34 and 35B were detected only in isolates from asymptomatic carriers, and serotypes 4, 7, 8, 14, and 20 were detected only in isolates from CAP carriers, which accounts for 15.7% and 7.2%, respectively. Overall, serotype 3 was the most common in both asymptomatic carriers and CAP carriers.

**Table 2:** Sequence types of 78 *S. pneumoniae* isolates among different serotypes

Serotype	Asymptomatic carriers		CAP carriers	
	n	Sequence types (n)	n	Sequence types (n)
3	12	876 (2), 230 (1), 2754 (1), 2771 (1), 3397 (1), 4112 (1), 6227 (1), 6542 (1), 13,377 (1), untyped (2)	9	271 (2), 320 (2), 902 (1), 4655 (1), 6327 (1), 7768 (1), 12,449 (1)
6	9	11,972 (2), 876 (1), 2754 (1), 3397 (1), 4112 (1), 4560 (1), 6209 (1), 13,610 (1)	1	180 (1)
19F	4	271 (3), 90 (1)	7	271 (5), 320 (1), untyped (1)
23A	3	230 (1), 6227 (2)	2	untyped (2)
35A/C	3	9120 (1), 10,236 (1), untyped (1)	1	13,862 (1)
35B	2	4112 (1), 9063 (1)	0	–
19A	2	320 (2)	3	271 (1), 320 (1), 1465 (1)
15B/C	2	3397 (2)	0	–
18	2	870 (2)	0	–
34	2	3397 (1), 11,964 (1)	0	–
11	1	6739 (1)	0	–
13	1	3397 (1)	0	–
22	1	6791 (1)	0	–
16F	1	6542 (1)	0	–
23B	1	2754 (1)	0	–
23F	1	14,604 (1)	2	81 (2)
4	0	–	1	505 (1)
7	0	–	1	untyped (1)
8	0	–	2	271 (1), 3500 (1)
14	0	–	1	271 (1)
20	0	–	1	180 (1)
Total	47		31	

### MLST analysis of *S. pneumoniae*

A total of 35 STs were identified in the 78 *serotypeable S. pneumoniae isolates*. The dominant STs were ST271 (13/78, 16.6%), ST320 (6/78, 7.7%) and ST3397 (6/78, 7.7%). 8 new STs were also detected, which were related to serotypes 3, 35, 23A, 19F, and 7. Most of isolates with ST271 were typed into serotype 19F, and all isolates with ST81 were typed into serotype 23F. Additionally, strains with ST320 were typed into serotypes 19A, 3, and 19F (Table 2).

**Fig. 1:**
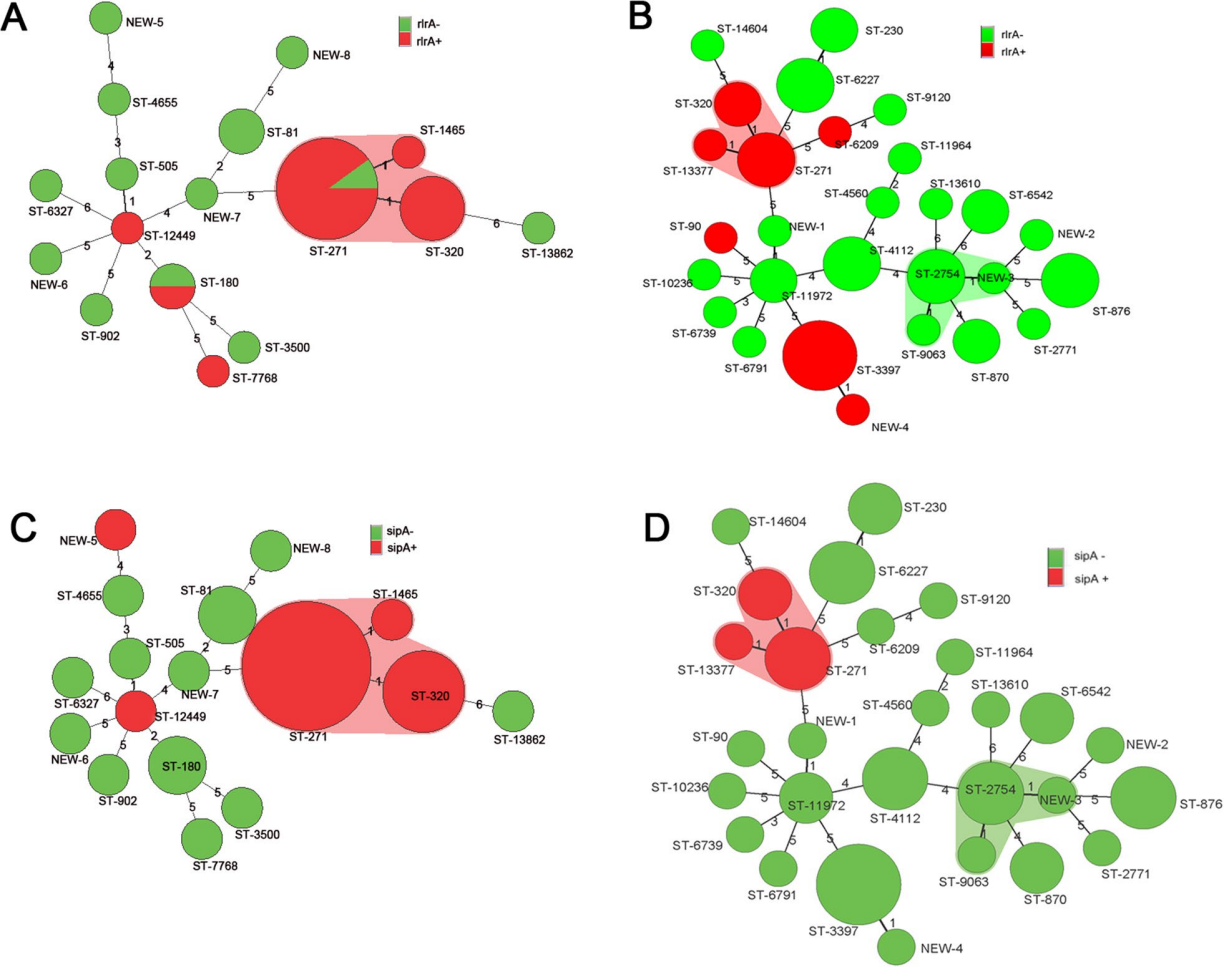
Minimum spanning tree (MST) analysis of strains based on the allelic profiles generated by MLST about pili genes. In the MST, the STs are displayed as circles. The size of each circle indicates the number of isolates within this particular type. The digits on the lines between two circles represent the differences in the numbers of the two types. With or without pili genes are represented by different colors. **A** Colors categorize with or without *rlrA* in CAP carriers; **B** colors categorize with or without *rlrA* in asymptomatic carriers. **C** Colors categorize with or without *sipA* in CAP carriers; **D** colors categorize with or without *sipA* in asymptomatic carriers

MST analysis based on allelic profiles showed that ST271 (n = 10) was the most common ST followed by ST320 (n = 4), ST81 (n = 2), and ST180 (n = 2) in the CAP carriers group. 17 STs were divided into 1 clonal complex 271 (CC271) and 14 singletons. CC271 contained 3 STs and 15 strains. ST271 was the founder ST of CC271 and contained 10 strains; the other 2 STs (ST320 and ST1465) of CC271 were single-locus variants (SLVs) of ST271 (Fig. 1A, C).

In the asymptomatic carriers group, MST results revealed that the major STs were ST876 (n = 5), ST3397 (n = 5), and ST230 (n = 4). 28 STs were divided into 2 CCs (CC271 and CC2754) and 28 singletons. CC271 contained 3 STs and 6 strains. ST271 was the founder ST of CC271 and contained 3 strains; the other 2 STs (ST320 and ST13377) of CC271 were SLVs of ST271. CC2754 contained 3 STs and 5 strains. ST2754 was the founder ST of CC2754 and contained 3 strains; the other 2 STs (ST9063 and ST NEW-3) of CC2754 were SLVs of ST2754 (Fig. 1B, D).

### Antimicrobial susceptibilities of *S. pneumoniae*

The antibiotic activities of 83 *S. pneumoniae* against the 17 antimicrobials were listed in Table 3. All isolates were susceptible to moxifloxacin (MOX). The resistance rates to erythromycin (ERY), clindamycin (CLI), tetracycline (TET) and trimethoprim-sulfamethoxazole (SXT) were 100%, 100%, 77.1% and 69.9%, respectively. The proportions of non-susceptible strains against TET, cefotaxime (CEF) and penicillin (PEN) were higher in the CAP carriers group, compared with asymptomatic carriers group (P < 0.05).

**Table 3:** Antimicrobial susceptibility of the 83 *S. pneumoniae* isolates to 17 antimicrobial agents

Antimicrobial agents^a^	Total (n = 83)			Asymptomatic carriers (n = 52)			CAP carriers (n = 31)			P value
	S^b^	I	R	S	I	R	S	I	R	
ERY	0 (0.0)	0 (0.0)	83 (100.0)	0 (0.0)	0 (0.0)	52 (100.0)	0 (0.0)	0 (0.0)	31 (100.0)	–
CLI	0 (0.0)	0 (0.0)	83 (100.0)	0 (0.0)	0 (0.0)	52 (100.0)	0 (0.0)	0 (0.0)	31 (100.0)	–
SXT	13 (15.7)	12 (14.5)	58 (69.9)	8 (15.4)	7 (13.5)	37 (71.2)	5 (16.1)	5 (16.1)	21 (67.7)	0.935
TET	13 (15.7)	6 (7.2)	64 (77.1)	12 (23.1)	2 (3.8)	38 (73.1)	1 (3.2)	4 (12.9)	26 (83.9)	0.014
CXM	38 (45.8)	10 (12.0)	35 (42.2)	27 (51.9)	8 (15.4)	17 (32.7)	11 (35.5)	2 (6.5)	18 (58.1)	0.065
CHL	75 (90.4)	0 ( (0.0)	8 (9.6)	47 (90.4)	0 (0.0)	5 (9.6)	28 (90.3)	0 (0.0)	3 (9.7)	0.993
CEF	68 (81.9)	5 (6.0)	10 (12.0)	47 (90.4)	1 (1.9)	4 (7.7)	21 (67.7)	4 (12.9)	6 (19.4)	0.028
PEN	24 (28.9)	38 (45.8)	21 (25.3)	15 (28.8)	29 (55.8)	8 (15.4)	9 (29.0)	9 (29.0)	13 (41.9)	0.015
CRO (Non-meningitis)	81 (97.6)	1 (1.2)	1 (1.2)	52 (100.0)	0 (0.0)	0 (0.0)	29 (93.5)	1 (3.2)	1 (3.2)	0.134
MER	74 (89.2)	8 (9.6)	1 (1.2)	47 (90.4)	4 (7.7)	1 (1.9)	27 (87.1)	4 (12.9)	0 (0.0)	0.475
CFP (Non-meningitis)	72 (86.7)	10 (12.0)	1 (1.2)	48 (92.3)	4 (7.7)	0 (0.0)	24 (77.4)	6 (19.4)	1 (3.2)	0.102
MOX	83 (100.0)	0 (0.0)	0 (0.0)	52 (100.0)	0 (0.0)	0 (0.0)	31 (100.0)	0 (0.0)	0 (0.0)	–
LEV	82 (98.8)	1 (1.2)	0 (0.0)	51 (98.1)	1 (1.9)	0 (0.0)	31 (100.0)	0 (0.0)	0 (0.0)	1.000
VAN	81 (97.6)	0 (0.0)	2 (2.4)	52 (100.0)	0 (0.0)	0 (0.0)	29 (93.5)	0 (0.0)	2 (6.5)	0.137
RIF	79 (95.2)	2 (2.4)	2 (2.4)	51 (98.1)	1 (1.9)	0 (0.0)	28 (90.3)	1 (3.2)	2 (6.5)	0.123
AMPC/CVA (Non-meningitis)	80 (96.4)	3 (3.6)	0 (0.0)	51 (98.1)	1 (1.9)	0 (0.0)	29 (93.5)	2 (6.5)	0 (0.0)	0.553
LIN	79 (95.2)	0 (0.0)	4 (4.8)	50 (96.2)	0 (0.0)	2 (3.8)	29 (93.5)	0 (0.0)	2 (6.5)	0.995

^a^*ERY* erythromycin, *CLI* clindamycin, *SXT* trimethoprim-sulfamethoxazole, *TET* tetracycline, *CXM* cefuroxime, *CHL* chloramphenicol, *CEF* cefotaxime, *PEN* penicillin, *CRO* ceftriaxone, *MER* meropenem, *CFP* cefepime, *MOX* moxifloxacin, *LEV* levofloxacin, *VAN* vancomycin, *RIF* rifampicin, *AMPC/CVA* amoxicillin/clavulanic acid, *LIN* linezolid

^b^*I* intermediate, *R* resistant, *S* susceptible

The resistance patterns of the pneumococcal isolates were shown in Table 4. About 90.4% (75/83) of the isolates were classified as MDR, and the most common resistance pattern was ERY-CLI-SXT-TET (21/83, 25.3%). To be specific, the most frequent pattern in the CAP carriers and asymptomatic carriers groups were ERY-CLI-TET and ERY-CLI-SXT-TET, respectively. Among the 75 MDR isolates, 3 (19/75, 25.3%), 19F (11/75, 14.7%), and 6 (7/75, 9.3%) were the most common serotypes. The most common resistance patterns of serotype 3 were ERY-CLI-SXT-TET and ERY-CLI-TET, and that of serotype 19F was ERY-CLI-SXT-TET-CXM-CEF-PEN.

**Table 4:** Antimicrobial resistance pattern of the 83 *S. pneumoniae* isolates

Resistance pattern	Total (n = 83)	Asymptomatic carriers (n = 52)		CAP carriers (n = 31)	
	n (%)	n (%)	Serotype (n)	n (%)	Serotype (n)
ERY-CLI	8 (9.6)	6 (11.5)	6 (2), 3 (2), 35 (1), untyped (1)	2 (6.5)	3 (1), 6 (1)
ERY-CLI-SXT	4 (4.8)	3 (5.8)	34 (1), 22 (1), 35 (1)	1 (3.2)	23F (1)
ERY-CLI-TET	10 (12.0)	3 (5.8)	3 (2),6 (1)	7 (22.6)	3 (2), 4 (1), 14 (1), 23F (1), 23A (1), 35 (1)
ERY-CLI-CXM	2 (2.4)	2 (3.8)	6 (1), untyped (1)	0 (0.0)	–
ERY-CLI-MER	1 (1.2)	1 (1.9)	3 (1)	0 (0.0)	–
ERY-CLI-LIN	1 (1.2)	1 (1.9)	6 (1)	0 (0.0)	–
ERY-CLI-SXT-TET	21 (25.3)	18 (34.6)	3 (4), 23A (3), 6 (2), 18 (2), 35 (2), 23B (1), 11 (1), untyped (3)	3 (9.7)	8 (2),19F (1)
ERY-CLI-TET-CEF	1 (1.2)	1 (1.9)	6 (1)	0 (0.0)	–
ERY-CLI-SXT-CEF	1 (1.2)	1 (1.9)	35 (1)	0 (0.0)	–
ERY-CLI-TET-CXM-CEF	1 (1.2)	1 (1.9)	16F (1)	0 (0.0)	–
ERY-CLI-SXT-TET-CEF	1 (1.2)	1 (1.9)	19F (1)	0 (0.0)	–
ERY-CLI-SXT-TET-CXM	11 (13.3)	6 (11.5)	15 (2), 34 (1), 13 (1), 3 (1), 6 (1)	5 (16.1)	3 (2),19F (1), 23F (1), 23A (1)
ERY-CLI-SXT-TET-CXM-PEN	8 (9.6)	3 (5.8)	19A (2), 23F (1)	5 (16.1)	19A (3), 19F (2)
ERY-CLI-SXT-TET-CXM-CEF-PEN	7 (8.4)	3 (5.8)	19F (2), 3 (1)	4 (12.9)	3 (2), 19F (2)
ERY-CLI-SXT-TET-CXM-CEF-PEN-LIN	1 (1.2)	1 (1.9)	19F (1)	0 (0.0)	–
ERY-CLI-SXT-TET-CXM-CHL-PEN-CEF-PEN	1 (1.2)	1 (1.9)	3 (1)	0 (0.0)	–
ERY-CLI-SXT-TET-PEN-VAN-RIF-LIN	1 (1.2)	0 (0.0)	–	1 (3.2)	3 (1)
ERY-CLI-SXT-CXM-CEF-PEN-CRO-CFP	1 (1.2)	0 (0.0)	–	1 (3.2)	20 (1)
ERY-CLI-SXT-TET-CXM-CHL-CEF-PEN	1 (1.2)	0 (0.0)	–	1 (3.2)	19F (1)
ERY-CLI-SXT-TET-CXM-CHL-PEN-VAN-RIF-LIN	1 (1.2)	0 (0.0)	–	1 (3.2)	3 (1)

^a^*PEN* penicillin, *CXM* cefuroxime, *CRO* ceftriaxone, *ERY* erythromycin, *CLI* clindamycin, *LEV* levofloxacin, *SXT* trimethoprim/sulfamethoxazole, *MXF* moxifloxacin, *VAN* vancomycin, *TET* tetracycline

### Distribution of non-susceptible strains in CC271 and other singletons

As shown in Table 5, the percentages of non-susceptible strains against CXM, CEF, MER and cefepime (CFP) in CC271 were more than other singletons between two groups (P < 0.05). Moreover, the percentages of strains non-susceptible to TET, AMPC/CVA and LIN in CC271 were slightly higher compared with other singletons between two groups, but no obvious differences were observed (P > 0.05).

**Table 5:** Differences on the distribution of strains that were non-susceptible to antibiotics in CC271 and the others

Antimicrobial agents^a^	Asymptomatic carriers (n = 52)			CAP carriers (n = 31)		
	CC271, n (%)	Others, n (%)	P values^b^	CC271, n (%)	Others, n (%)	P values^b^
ERY	6 (100)	46 (100)	–	15 (100)	6 (100)	–
CLI	6 (100)	46 (100)	–	15 (100)	6 (100)	–
SXT	6 (100)	38 (82.6)	0.573	15 (100)	6 (100)	0.043
TET	6 (100)	34 (73.9)	0.316	15 (100)	6 (100)	1.000
CXM	6 (100)	19 (41.3)	0.002	14 (93.3)	6 (100)	0.009
CHL	0 (0.0)	5 (10.9)	1.000	2 (13.3)	0 (0.0)	0.600
CEF	4 (66.7)	1 (2.2)	0.000	8 (53.3)	4 (66.7)	0.015
PEN	6 (100)	31 (67.4)	0.165	14 (93.3)	6 (100)	0.015
CRO (Non-meningitis)	0 (0.0)	0 (0.0)	–	0 (0.0)	0 (0.0)	0.484
MER	3 (50.0)	2 (4.4)	0.043	4 (26.7)	3 (50.0)	0.008
CFP (Non-meningitis)	4 (66.7)	0 (0.0)	0.037	6 (40.0)	4 (66.7)	0.000
MOX	0 (0.0)	0 (0.0)	–	0 (0.0)	0 (0.0)	–
LEV	0 (0.0)	1 (2.2)	1.000	0 (0.0)	0 (0.0)	–
VAN	0 (0.0)	0 (0.0)	–	2 (13.3)	0 (0.0)	0.226
RIF	0 (0.0)	1 (2.2)	1.000	3 (0.2)	0 (0.0)	0.101
AMPC/CVA (Non-meningitis)	1 (16.7)	0 (0.0)	0.115	1 (6.7)	1 (16.7)	1.000
LIN	1 (16.7)	1 (2.2)	0.219	2 (13.3)	1 (16.7)	0.226

^a^*ERY* erythromycin, *CLI* clindamycin, *SXT* trimethoprim-sulfamethoxazole, *TET* tetracycline, *CXM* cefuroxime, *CHL* chloramphenicol, *CEF* cefotaxime, *PEN* penicillin, *CRO* ceftriaxone, *MER* meropenem, *CFP* cefepime, *MOX* moxifloxacin, *LEV* levofloxacin, *VAN* vancomycin, *RIF* rifampicin, *AMPC/CVA* amoxicillin/clavulanic acid, *LIN* linezolid

^b^Fisher’s Exact Test was used

### Virulence/pili genes analysis of *S. pneumoniae*

Among the 52 isolates from asymptomatic carriers, all isolates carried *lytA*, *cbpG* and *pavA*. Additionally, there were 96.2%, 98.1%, 94.2% isolates carrying *ply*, *pspA* and *cbpA*. In all isolates from CAP carriers, *ply, lytA, pspA, cbpA, cbpG* and *pavA* were detected. However, *rlrA* and *sipA* genes were amplified in only 37.3% and 27.7% of *S. pneumoniae*. The percentages of isolates carrying *rlrA and sipA* in CAP carriers group were higher than those of asymptomatic carriers group (P < 0.05), indicating significant association between the genes of *rlrA* and *sipA* and the source of *S. pneumoniae* (Table 6).

**Table 6:** Distribution of virulence/pili genes in asymptomatic carriers and CAP carriers

Virulence/pili genes	Total (n = 83)	Asymptomatic carriers (n = 52)	CAP carriers (n = 31)	P value
*ply* (n, %)	81 (97.6)	50 (96.2)	31 (100.0)	0.526
*lytA* (n, %)	83 (100.0)	52 (100.0)	31 (100.0)	–
*pspA* (n, %)	82 (98.8)	51 (98.1)	31 (100.0)	1.000
*pavA* (n, %)	83 (100.0)	52 (100.0)	31 (100.0)	–
*cbpA* (n, %)	80 (96.4)	49 (94.2)	31 (100.0)	0.289
*cbpG* (n, %)	83 (100.0)	52 (100.0)	31 (100.0)	–
*rlrA* (n, %)	31 (37.3)	14 (26.9)	17 (54.8)	0.011
*sipA* (n, %)	23 (27.7)	6 (11.5)	17 (54.8)	0.000

Additionally, we observed that the most predominant serotype carrying *sipA* was 19F (n = 9), followed by 3 (n = 6) and 19A (n = 5). Similarly, the order of serotypes carrying both *rlrA* and *sipA* was 19F (n = 8), 3 (n = 6) and 19A (n = 5). Moreover, 95.2% and 100% of the isolates in CC271 carried *rlrA* and *sipA* (Fig. 1).

## Discussion

The current study was the first to evaluate the serotypes distribution, virulence/pili genes, and antibiotic susceptibility of *S. pneumoniae* from healthy asymptomatic participants and CAP patients in China. We found that the most frequent serotypes were 3 in CAP carriers and asymptomatic carriers. Additionally, 4 serotypes exclusively associated with CAP carriers, and 7 with asymptomatic carriers were observed. Over half of the isolates belonged to PCV13 serotypes and were resistant to ERY, CLI, SXT, and TET. The MDR rate was up to 90.2%. The dominant STs were ST271, ST320, and ST3397, respectively. The most interesting findings were significant association between the *rlrA* and *sipA* genes and the source of *S. pneumoniae*.

It is well known that the distribution of *S. pneumoniae* serotypes changes over age, geographic region, time, and diseases [27, 28]. In our study, the most frequent serotypes in CAP carriers group were 3, 19F, 19A, which were similar to other recent studies in China [16, 29], however different with serotypes in Italy [30]. Additionally, the major serotypes from asymptomatic carriers were 3, 6, and 19F in our study, whereas were 19F, 6B, and 6C in Brazil [31], indicating that serotypes is closely related to geographic region. NT isolates are deemed to be less virulent, due to lacking the polysaccharide capsule [32]. Correspondingly, previous study has analyzed the distribution of NT isolates between the asymptomatic carriers and IPDs, and found NT isolates were only from asymptomatic carriers [31]. Similarly, NT isolates were only detected from asymptomatic carriers in our study as well. On the other side, we found that serotypes 3 and 19F were frequently found in both asymptomatic carriers and CAP carriers. Indeed, these serotypes are common in asymptomatic carriers and CAP carriers worldwide [16, 33–35].

PCVs can effectively reduce the burden of pneumococcal diseases and improve the population immunity [36]. Previous studies have revealed that incidence of vaccine-covered serotypes was significantly decreased after vaccination with PCVs. In parallel, occurrence of non-PCVs serotypes was increased over time [37, 38]. In our study, the overall coverage rate of PCV13 was 66.3%, which was conformed to previous study reported the 50–68% coverage of PCV13 in mainland China [39]. Additionally, we also found that the major serotypes in CAP carriers and asymptomatic carriers were PCV13 serotypes. Those results indicated that PCV13 may be useful to prevent against *S. pneumoniae*. However, several studies have concluded the different points regarding the PCV13 efficacy for serotype 3 in different regions. For example, Morre et al. [40] have reported that PCV13 presents high effectiveness against pneumococcal disease in USA. On the contrary, Andrews et al. [41] have stated that PCV13 is effective for most serotypes except for serotype 3 in UK. Additionally, a similar study in Hong Kong, China has found that the efficacy of PCV13 against pneumococcal disease caused by serotype 3 is low [42]. Therefore, whether the introduction of PCV13 against pneumococcal disease caused by serotype 3 is effective needs to be monitored, because serotype 3 was the most common in both asymptomatic carriers and CAP carriers.

The increase of antibiotic resistance enables the *S. pneumoniae* to be a worrying threat for public health. Previous studies have demonstrated that overuse of antibiotic contributes to the increase of antibiotic resistance [43, 44]. Although many measures have been taken to regulate the use of antibiotics in hospitals, antibiotics use is also widespread in China. A previous report has shown that duration of bacterial carriage is closely associated with prevalence of resistance [45]. In our study, higher resistance rates to CEF, TET and PEN in CAP carriers compared with asymptomatic carriers were shown. This may be related to the differences in serotype distribution and duration of bacterial carriage. Additionally, a few pneumococcal isolates in asymptomatic carriers and CAP carriers groups were susceptible to other antibiotics, such as CHL, CEF, CRO, MER, CFR, MOX, LEV, VAN, RIF, AMPC/CVA, and LIN, which brought hope to the treatment of drug-resistant *S. pneumoniae*. Therefore, rational use of antibiotics may be appropriate measure to control the spread of antibiotic-resistant strains.

The most common STs in this study were ST271 and ST320, which was similar to the findings of other reports in other regions of China [46, 47]. The predominant CC was CC271 in our study, which it was found in both CAP carriers and asymptomatic carriers. Additionally, CC271 was also closely associated with the carriage of *rlrA* and *sipA*. Consistent with our study, previous study has revealed that the PI-1 and PI-2 encoded by *rlrA* and *sipA* genes are present in isolates of CC271 [48]. In the current study, the carriage rates of *rlrA* and *sipA* genes from CAP carriers were higher than those of asymptomatic carriers, indicating that *rlrA* and *sipA* may be closely associated with high virulence. Additionally, our study showed that the genes including *ply, lytA, pspA, cbpA, cbpG* and *pavA* were widely conserved, suggesting that these may be candidates for developing vaccine in future. Moreover, we found the *rlrA* was present in serotypes 19F, 3, 19A, which has also been reported in previous study [26]. However, it has been reported that *sipA* gene is present in over half of pneumococcal isolates and commonly detected in serotypes 19F, 11A and 7A/F [26]. In this study, the most dominant serotype carrying *sipA* was 19F, followed by 3 and 19A. Taken together, *rlrA and sipA* may be important for colonization and pathogenicity of pneumococcal isolates.

The limitations of this study should be noted. First, our surveillance was not a population-based study but rather an actively participating surveillance, which led to the small numbers of isolates collected. All study populations have not been vaccinated with PCVs, and the changes after introduction of PCVs in this population remain unclear. Last, serogroup 6 was not typed, due to the limited laboratory equipment and reagents.

## Conclusions

The current study showed the serotype distribution and antimicrobial resistance of pneumococcal isolates from CAP carriers and asymptomatic carriers in Sichuan, China. In addition, high prevalence of MDR and high coverage of PCV13 emphasized the importance of rational use of antibiotics and the necessity to expand the immunization with PCV13. Importantly, some virulence/pili genes were conserved, which may supply new guidance for pneumococcal vaccines in the future.

## Data Availability

All data generated or analyzed during this study are included in this article.

## Abbreviations

*S. pneumoniae*: *Streptococcus pneumoniae*
CAP: Community-acquired pneumonia
IPDs: Invasive pneumococcal diseases
Non-IPDs: Non-invasive pneumococcal diseases
PCV: Pneumococcal conjugate vaccine
MDR: Multidrug resistant
STs: Sequence types
CC: Clonal complex
MST: Minimum spanning trees
SLVs: Single-locus variants
PCR: Polymerase chain reaction
NT: Non-typeable
MLST: Multilocus sequence typing
MICs: Minimum inhibitory concentrations
CLSI: Clinical and Laboratory Standards Institute

## References

[1] Ewig S, Birkner N, Strauss R, Schaefer E, Pauletzki J, Bischoff H (2009). New perspectives on community-acquired pneumonia in 388 406 patients. Results from a nationwide mandatory performance measurement programme in healthcare quality. Thorax.

[2] Rudan I, O’brien KL, Nair H, Liu L, Theodoratou E, Qazi S (2013). Epidemiology and etiology of childhood pneumonia in 2010: estimates of incidence, severe morbidity, mortality, underlying risk factors and causative pathogens for 192 countries. J Glob Health.

[3] File TM (2004). *Streptococcus pneumoniae* and community-acquired pneumonia: a cause for concern. Am J Med.

[4] Morozumi M, Chiba N, Okada T, Sakata H, Matsubara K, Iwata S (2013). Antibiotic susceptibility in relation to genotype of *Streptococcus pneumoniae*, *Haemophilus influenzae*, and *Mycoplasma pneumoniae* responsible for community-acquired pneumonia in children. J Infect Chemother.

[5] Schmitz J, Van Der Linden M, Al-Lahham A, Levina N, Pletz MW, Imöhl M (2017). Fluoroquinolone resistance in *Streptococcus pneumoniae* isolates in Germany from 2004–2005 to 2014–2015. Int J Med Microbiol.

[6] Korona-Glowniak I, Maj M, Siwiec R, Niedzielski A, Malm A (2016). Molecular epidemiology of *Streptococcus pneumoniae* isolates from children with recurrent upper respiratory tract infections. PLoS ONE.

[7] Kim SH, Song JH, Chung DR, Thamlikitkul V, Yang Y, Wang H (2012). Changing trends in antimicrobial resistance and serotypes of *Streptococcus pneumoniae* isolates in Asian countries: an Asian Network for Surveillance of Resistant Pathogens (ANSORP) study. Antimicrob Agents Chemother.

[8] Geno KA, Gilbert GL, Song JY, Skovsted IC, Klugman KP, Jones C (2015). Pneumococcal capsules and their types: past, present, and future. Clin Microbiol Rev.

[9] Kamerling JP. Pneumococcal polysaccharides: a chemical view. *Streptococcus pneumoniae*: molecular biology and mechanisms of disease. 1999: 81–114.

[10] Bentley SD, Aanensen DM, Mavroidi A, Saunders D, Rabbinowitsch E, Collins M (2006). Genetic analysis of the capsular biosynthetic locus from all 90 pneumococcal serotypes. PLoS Genet.

[11] Hu Y, Luo S, Tang X, Lou L, Chen Y, Guo J (2015). Comparative assessment of immunization coverage of migrant children between national immunization program vaccines and non-national immunization program vaccines in East China. Hum Vaccin Immunother.

[12] Kim SH, Chung DR, Song JH, Baek JY, Thamlikitkul V, Wang H (2020). Changes in serotype distribution and antimicrobial resistance of *Streptococcus pneumoniae* isolates from adult patients in Asia: emergence of drug-resistant non-vaccine serotypes. Vaccine.

[13] Hu J, Sun X, Huang Z, Wagner AL, Carlson B, Yang J (2016). *Streptococcus pneumoniae* and Haemophilus influenzae type b carriage in Chinese children aged 12–18 months in Shanghai, China: a cross-sectional study. BMC Infect Dis.

[14] Xie N, Chen ZY, Chen T, Zhu BQ, Xu L, Gao Y (2018). A cross-sectional survey assessing carriage of *Streptococcus pneumoniae* in a healthy population in Xinjiang Uygur autonomous region of China. Biomed Environ Sci.

[15] Bogaert D, De Groot R, Hermans PW (2004). *Streptococcus pneumoniae* colonisation: the key to pneumococcal disease. Lancet Infect Dis.

[16] Liang Z, Fu J, Li L, Yi R, Xu S, Chen J (2021). Molecular epidemiology of *Streptococcus pneumoniae* isolated from pediatric community-acquired pneumonia in pre-conjugate vaccine era in Western China. Ann Clin Microbiol Antimicrob.

[17] Li MC, Wang Y (2021). Serotype distribution and clinical characteristics associated with *Streptococcus pneumoniae* among Chinese children and adults with invasive pneumococcal disease: a multicenter observational study. Hum Vaccin Immunother.

[18] Pai R, Gertz RE, Beall B (2006). Sequential multiplex PCR approach for determining capsular serotypes of *Streptococcus pneumoniae* isolates. J Clin Microbiol.

[19] Sørensen UB (1993). Typing of pneumococci by using 12 pooled antisera. J Clin Microbiol.

[20] Enright MC, Spratt BG (1998). A multilocus sequence typing scheme for *Streptococcus pneumoniae*: identification of clones associated with serious invasive disease. Microbiology (Reading).

[21] Clinical and Laboratory Standards Institute (2018) Performance standards for antimicrobial susceptibility testing; twenty-eighth informational supplement. M100-S28. Clinical and Laboratory Standards Institute, Wayne.

[22] Mitchell AM, Mitchell TJ (2010). *Streptococcus pneumoniae*: virulence factors and variation. Clin Microbiol Infect.

[23] Khan F, Khan MA, Ahmed N, Khan MI, Bashir H, Tahir S (2018). Molecular characterization of pneumococcal surface protein A (PspA), serotype distribution and antibiotic susceptibility of *Streptococcus pneumoniae* strains isolated from Pakistan. Infect Dis Ther.

[24] Holmes AR, Mcnab R, Millsap KW, Rohde M, Hammerschmidt S, Mawdsley JL (2001). The pavA gene of *Streptococcus pneumoniae* encodes a fibronectin-binding protein that is essential for virulence. Mol Microbiol.

[25] Gosink KK, Mann ER, Guglielmo C, Tuomanen EI, Masure HR (2000). Role of novel choline binding proteins in virulence of *Streptococcus pneumoniae*. Infect Immun.

[26] Shakrin NN, Masri SN, Taib NM, Nordin SA, Jamal F, Desa MN (2014). Genotypic characterization of Malaysian human isolates of *Streptococcus pneumoniae* from carriage and clinical sources. Comp Immunol Microbiol Infect Dis.

[27] Saha SK, Naheed A, El Arifeen S, Islam M, Al-Emran H, Amin R (2009). Surveillance for invasive *Streptococcus pneumoniae* disease among hospitalized children in Bangladesh: antimicrobial susceptibility and serotype distribution. Clin Infect Dis.

[28] Wang X, Cong Z, Huang W, Li C (2020). Molecular characterization of *Streptococcus pneumoniae* isolated from pediatric patients in Shanghai. China Pediatr Pulmonol.

[29] Isturiz RE, Ramirez J, Self WH, Grijalva CG, Counselman FL, Volturo G (2019). Pneumococcal epidemiology among us adults hospitalized for community-acquired pneumonia. Vaccine.

[30] Di Pasquale M, Aliberti S, Azzari C, Moriondo M, Nieddu F, Blasi F (2017). Serotypes and antibiotic susceptibility of *Streptococcus pneumoniae* isolated from hospitalized patients with community-acquired pneumonia in Italy. SAGE Open Med.

[31] Pinto TCA, Neves FPG, Souza ARV, Oliveira LMA, Costa NS, Castro LFS (2019). Evolution of penicillin non-susceptibility among *Streptococcus pneumoniae* isolates recovered from asymptomatic carriage and invasive disease over 25 years in Brazil, 1990–2014. Front Microbiol.

[32] Sharma D, Baughman W, Holst A, Thomas S, Jackson D, Da Gloria CM (2013). Pneumococcal carriage and invasive disease in children before introduction of the 13-valent conjugate vaccine: comparison with the era before 7-valent conjugate vaccine. Pediatr Infect Dis J.

[33] Ziane H, Manageiro V, Ferreira E, Moura IB, Bektache S, Tazir M (2016). Serotypes and antibiotic susceptibility of *Streptococcus pneumoniae* isolates from invasive pneumococcal disease and asymptomatic carriage in a pre-vaccination period, in Algeria. Front Microbiol.

[34] Sulikowska A, Grzesiowski P, Sadowy E, Fiett J, Hryniewicz W (2004). Characteristics of *Streptococcus pneumoniae*, *Haemophilus influenzae*, and *Moraxella catarrhalis* isolated from the nasopharynges of asymptomatic children and molecular analysis of *S. pneumoniae* and *H. influenzae* strain replacement in the nasopharynx. J Clin Microbiol.

[35] Isozumi R, Ito Y, Ishida T, Hirai T, Ito I, Maniwa K (2008). Molecular characteristics of serotype 3 *Streptococcus pneumoniae* isolates among community-acquired pneumonia patients in Japan. J Infect Chemother.

[36] Oishi K, Tamura K, Akeda Y (2014). Global control of pneumococcal infections by pneumococcal vaccines. Trop Med Health.

[37] Richter SS, Diekema DJ, Heilmann KP, Dohrn CL, Riahi F, Doern GV (2014). Changes in pneumococcal serotypes and antimicrobial resistance after introduction of the 13-valent conjugate vaccine in the United States. Antimicrob Agents Chemother.

[38] Ubukata K, Chiba N, Hanada S, Morozumi M, Wajima T, Shouji M (2015). Serotype changes and drug resistance in invasive pneumococcal diseases in adults after vaccinations in children, Japan, 2010–2013. Emerg Infect Dis.

[39] Chen K, Zhang X, Shan W, Zhao G, Zhang T (2018). Serotype distribution of *Streptococcus pneumoniae* and potential impact of pneumococcal conjugate vaccines in China: a systematic review and meta-analysis. Hum Vaccin Immunother.

[40] Moore MR, Link-Gelles R, Schaffner W, Lynfield R, Holtzman C, Harrison LH (2016). Effectiveness of 13-valent pneumococcal conjugate vaccine for prevention of invasive pneumococcal disease in children in the USA: a matched case-control study. Lancet Respir Med.

[41] Andrews NJ, Waight PA, Burbidge P, Pearce E, Roalfe L, Zancolli M (2014). Serotype-specific effectiveness and correlates of protection for the 13-valent pneumococcal conjugate vaccine: a postlicensure indirect cohort study. Lancet Infect Dis.

[42] Ho PL, Law PY, Chiu SS (2019). Increase in incidence of invasive pneumococcal disease caused by serotype 3 in children eight years after the introduction of the pneumococcal conjugate vaccine in Hong Kong. Hum Vaccin Immunother.

[43] Cillóniz C, Ardanuy C, Vila J, Torres A (2016). What is the clinical relevance of drug-resistant pneumococcus?. Curr Opin Pulm Med.

[44] Messina NL, Williamson DA, Robins-Browne R, Bryant PA, Curtis N (2020). Risk factors for carriage of antibiotic-resistant bacteria in healthy children in the community: a systematic review. Pediatr Infect Dis J.

[45] Lehtinen S, Blanquart F, Croucher NJ, Turner P, Lipsitch M, Fraser C (2017). Evolution of antibiotic resistance is linked to any genetic mechanism affecting bacterial duration of carriage. Proc Natl Acad Sci U S A.

[46] Li L, Fu J, Li S, Guo D, Chen Z, Chen S (2018). Phenotypic and molecular characterization of *Streptococcus pneumoniae* in pre-conjugate vaccine era: a Chinese hospital-based retrospective study. Vaccine.

[47] Shi W, Li J, Dong F, Qian S, Liu G, Xu B (2019). Serotype distribution, antibiotic resistance pattern, and multilocus sequence types of invasive *Streptococcus pneumoniae* isolates in two tertiary pediatric hospitals in Beijing prior to PCV13 availability. Expert Rev Vaccines.

[48] Zähner D, Gudlavalleti A, Stephens DS (2010). Increase in pilus islet 2-encoded pili among *Streptococcus pneumoniae* isolates, Atlanta, Georgia, USA. Emerg Infect Dis.

